# Paediatric cyclical Cushing’s disease due to corticotroph cell hyperplasia

**DOI:** 10.1186/s12902-015-0024-3

**Published:** 2015-06-12

**Authors:** E. Noctor, S. Gupta, T. Brown, M. Farrell, M. Javadpour, C. Costigan, A. Agha

**Affiliations:** Department of Endocrinology and Neurosurgery, Beaumont Hospital, Beaumont Road, Dublin 9, Ireland; Department of Neuropathology, Beaumont Hospital, Dublin, Ireland; Department of Neurosurgery, Beaumont Hospital, Dublin, Ireland; Department of Paediatric Endocrinology, Our Lady’s Hospital for Sick Children, Dublin, Ireland

**Keywords:** Corticotroph hyperplasia, Cyclical hypercortisolism, Paediatric Cushing’s

## Abstract

**Background:**

Cushing’s disease is very rare in the paediatric population. Although uncommon, corticotroph hyperplasia causing Cushing’s syndrome has been described in the adult population, but appears to be extremely rare in children. Likewise, cyclical cortisol hypersecretion, while accounting for 15 % of adult cases of Cushing’s disease, has only rarely been described in the paediatric population. Here, we describe a very rare case of a 13-year old boy with cyclical cortisol hypersecretion secondary to corticotroph cell hyperplasia.

**Case presentation:**

The case is that of a 13-year old boy, presenting with a long history of symptoms and signs suggestive of hypercortisolism, who was found to have cyclical ACTH-dependent hypercortisolism following dynamic pituitary testing and serial late-night salivary cortisol measurements. The patient underwent endoscopic transsphenoidal resection of the pituitary. Early surgical remission was confirmed by undetectable post-operative morning plasma cortisol levels. Histology and immunocytochemistry of the resected pituitary tissue showed extensive corticotroph cell hyperplasia.

**Conclusion:**

This report describes a rare case of cyclical Cushing’s disease secondary to corticotroph hyperplasia in a paediatric patient. This highlights the challenging and varied nature of Cushing’s disease and its diagnosis, and the need to keep a differential diagnosis in mind during the diagnostic process.

## Background

Cushing’s disease is rare in the paediatric population, and is invariably due to a corticotroph adenoma, usually a microadenoma [1]. Corticotroph hyperplasia as a cause of Cushing’s syndrome, although uncommon, has been described in the adult population. However, this appears to be extremely rare in children. Also, cyclical cortisol hypersecretion, while accounting for 15 % of adult cases of Cushing’s disease [2], has very rarely been described in the paediatric population [3]. Here we describe the case of a 13-year old boy with cyclical cortisol hypersecretion secondary to corticotroph cell hyperplasia, and review the relevant literature with regard to paediatric Cushing’s disease.

## Case presentation

A 13 year-old boy was referred to the endocrine service for evaluation. Our patient and his family described a history of early adrenarche aged 5, and, beginning at age 9, the gradual onset of marked weight gain and development of a rounded, plethoric facies. In retrospect, proximal muscle weakness was also felt to have begun at this time. He presented to his primary care practitioner at the age of 12, and was referred on to a paediatric endocrinology tertiary referral centre for further assessment.

**Fig 1:**
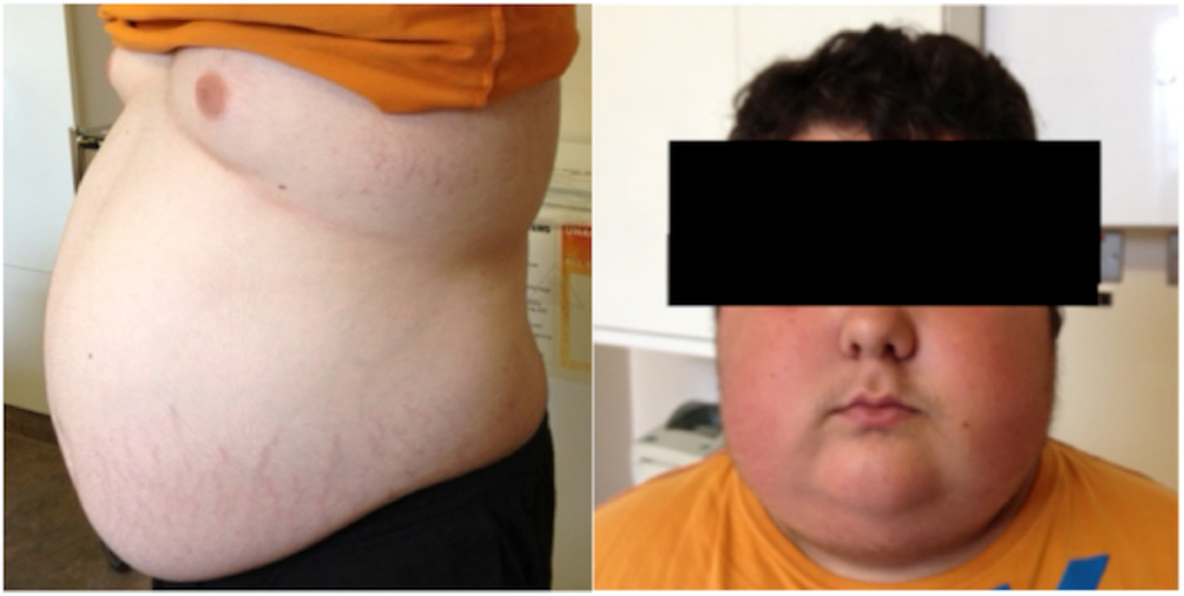
Pictures taken the week prior to surgery, demonstrating increased abdominal circumference, striae, and Cushingoid facies

At initial assessment, weight was 95.9 kg (above the 99.6^th^ centile) with a height of 147 cm (between 10- 25^th^ centile). He had markedly Cushingoid facies, an interscapular fat pad, and increased abdominal girth with striae, with objective evidence of proximal myopathy (Fig. 1). Blood pressure was normal.

A 1 mg overnight dexamethasone suppression revealed a post-suppression cortisol value of 258 nmol/L (normal <50 nmol/l). Twenty-four hour urinary free cortisol was markedly elevated at 987 nmol/24 h (reference range 0–83 nmol/24 h). Given the characteristic clinical picture, and 2 positive screening tests, a diagnosis of Cushing’s syndrome was made. His ACTH level was not suppressed, consistent with ACTH-dependent Cushing’s syndrome. A peripheral human corticotropin-releasing hormone (CRH) test showed a greater than 50 % rise in ACTH after 15 min, (60.1 pg/ml at 0 mins rising to 104 pg/ml at 15 mins), along with an almost 50 % rise in cortisol (756 to 1126 nmol/L). This is highly characteristic of pituitary dependent Cushing’s syndrome (Cushing’s disease) [4]. A contrast-enhanced dynamic pituitary MRI scan was performed, and interpreted by a specialist pituitary neuroradiologist. This revealed a radiologically normal pituitary gland.

**Fig 2:**
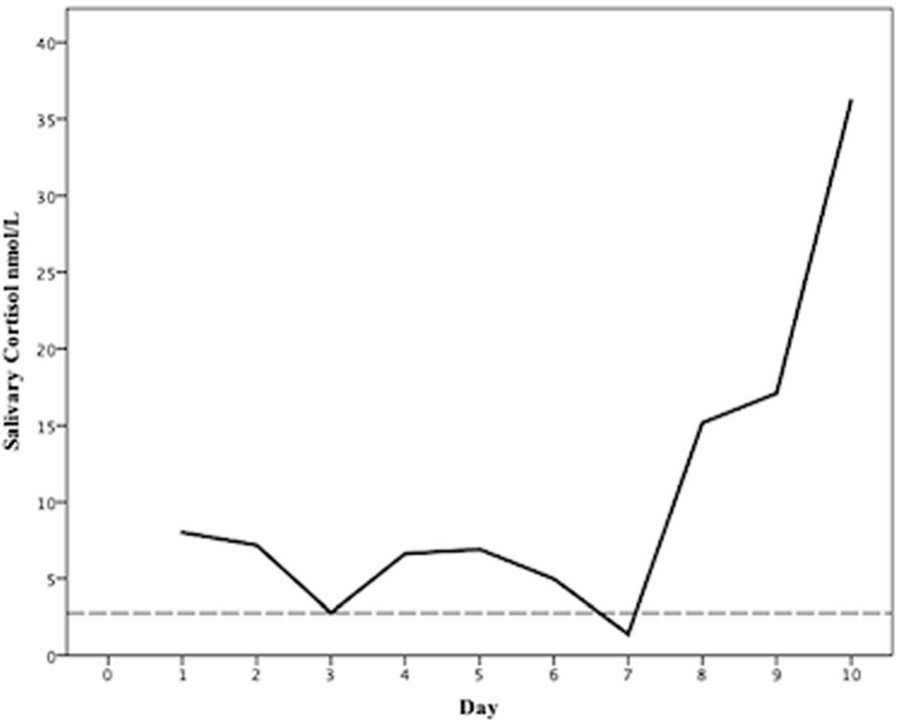
Late-night salivary cortisol over 10 consecutive days in a boy with suspected Cushing’s disease. Dashed line represents upper limit of normal for salivary cortisol (2.5 nmol/L)

On questioning, the patient reported fluctuations in his symptoms with some days feeling more symptomatic than others. To assess for the possibility of cyclical oversecretion of ACTH/cortisol, we performed serial salivary cortisol measurement, which has been shown to have similar sensitivity to urine sampling for the detection of cyclical hypercortisolism [5] (the patient was not taking any medication that could interfere with the result). This showed several peaks and two normal value troughs (Fig 2), consistent with cyclical Cushing’s disease.

To confirm the diagnosis of pituitary dependant Cushing’s syndrome, inferior petrosal sinus sampling (IPSS) was performed under general anaesthesia with samples obtained at baseline, and then at different time points following CRH administration. Unfortunately, the first test showed no central to peripheral gradient as it was done on a day when the disease was not active. A repeat IPSS was subsequently performed on a morning when the disease was active - an early morning cortisol on the morning of the procedure was 845 nmol/l, confirming hypercortisolaemia. This second IPSS showed a marked central to peripheral gradient (Left petrosal ACTH concentration at 0 mins > 2100 pg/ml, left peripheral ACTH concentration 167 pg/ml at 0 mins). A marked left to right gradient was also evident (Results summarised in Table 1). This confirmed that the patient had Cushing’s disease.

**Table 1:** Summary of dynamic tests

Test	Result	Interpretation
1 mg overnight DST	Morning cortisol 258 nmol/L	Failure to suppress cortisol secretion
	(normal suppression < 50 nmol/L)	
24 h UFC	987 nmol/L	Values greater than 3 times ULN are highly suggestive of Cushing’s syndrome
	(Ref. 0–83 nmol/L)	
CRH test	ACTH 0 mins 60 pg/ml	Greater than 50 % rise in ACTH, suggestive of Cushing’s syndrome
	Cortisol 0 mins 756 nmol/L	
	ACTH 15 mins 104 pg/ml	
	Cortisol 15 mins 1126 nmol/L	
IPSS (CRH augmented)	ACTH values 5 mins post CRH	No central to peripheral gradient
	Left petrosal 166 pg/ml	
	Right petrosal 92 pg/ml	
	peripheral 77 pg/ml	
IPSS (CRH augmented)	ACTH values 5 mins post CRH	Marked central to peripheral gradient (PPV 98 %)
	Left petrosal >2100 pg/ml	
	Right petrosal 271 pg/ml	Lateralising to left (accuracy 69 %) [28, 29]
	peripheral 176 pg/ml	

DST- Dexamethasone suppression test

UFC – Urinary free cortisol

CRH – Corticotropin-releasing hormone

ACTH- Adrenocorticotrophic hormone

IPSS – Inferior petrosal sinus sampling

ULN - Upper limit of normal

PPV-positive predictive value for diagnosis of pituitary-dependant Cushing’s syndrome (Cushing’s disease)

The patient underwent endoscopic transsphenoidal exploration of the pituitary by our dedicated pituitary surgeon. This revealed abnormal soft tissue in the midline and to the left of the pituitary fossa, all of which was macroscopically removed at surgery. Aside from transient diabetes insipidus, our patient’s post-operative course was unremarkable. Several serial morning (8 am) serum cortisol levels were all less than 28 nmol/l confirming early remission (defined as post-operative cortisol values < 50 nmol/L). The patient was discharged on hydrocortisone 10 mgs twice daily.

### Histology and immunostaining

**Fig 3:**
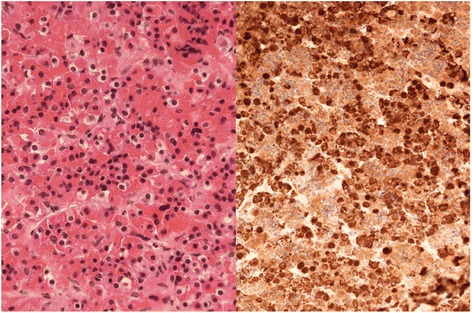
Resected adenohypophysis. Cells with eosinophilic cytoplasm and clear cytoplasm with occasional cells having basophilic cytoplasm are seen on the left. The specimen on the right shows extensive ACTH staining without evidence of focal suppression

The excised surgical specimen consisted of adenohypophysis characterised by an admixture of cells arranged into nests (Fig 3). Cells with eosinophilic cytoplasm and clear cytoplasm, together with occasional cells having basophilic cytoplasm were all present. Immunocytochemistry demonstrated immunoreactivity for TSH, FSH, LH, and prolactin. Additionally there was extensive widespread ACTH expression throughout all of the resected material. There was no evidence of isolated focal ACTH immunostaining in the resected pituitary tissue. ACTH immunostaining would have been markedly suppressed in the non-adenomatous gland in the presence of an ACTH-secreting pituitary adenoma if such an adenoma was present in the gland, but not in the laboratory specimen (a phenomenon which is recognised in some cases following successful neurosurgery for Cushing’s disease). Also, reticulin staining demonstrated a normal pericellular reticulin pattern, as opposed to the pattern of reticulin disruption that would be expected if an adenoma were present [6]. Therefore, this extensive ACTH expression in the resected adenohypophysis tissue reflected diffuse corticotroph cell hyperplasia.

### Clinical course

Six weeks post-operatively, the patient underwent a glucagon stimulation test, which showed severe growth hormone deficiency and persistent severe hypocortisolaemia. The patient was started on recombinant human growth hormone (GH) therapy. He later developed post-GH secondary hypothyroidism and was treated with L-thyroxine. One year later, he remained hypocortisolaemic, and reported excellent progress. His weight has declined from a peak of 97 to 63 kg and his height has increased from 147 to 152 cm. His Cushingoid features have also markedly improved.

## Conclusion

Pituitary dependent Cushing’s syndrome is rare in the paediatric population and is invariably due to corticotroph adenomas (1). In the adult population, multifocal corticotroph hyperplasia has been shown to precede adenoma development [7], and even to co-exist with adenomas [8–10]. However, several paediatric series have been reported – the largest by far a 28-year prospective study in the National Institutes of Health, describing 200 children [11], none of whom demonstrated evidence of corticotroph hyperplasia as the aetiology of their Cushing’s disease. The other large case series are all considerably smaller by comparison, for example; a 15-year retrospective study from the Mayo Clinic reporting only 22 patients [12], 33 cases described in the southern United States [13], and 25 cases described in Britain [14]. However, none of the paediatric patients in these case series demonstrated corticotroph hyperplasia as the aetiology of their Cushing’s disease. However, corticotroph hyperplasia as a cause of Cushing’s disease in a paediatric patient was reported in a single case from Finland [15]. Surprisingly, and in sharp contrast with the American and British series, 3 of 15 patients in a Brazilian case series [16] were reported as having increased numbers of ACTH staining cells with no evidence of an adenoma but no detailed histology was presented (10).

While the absence of an adenoma from the surgical specimen following successful transsphenoidal surgery for Cushing’s disease is sometimes seen [11], in this case, the normal adenohypophysis should show marked suppression of ACTH immunostaining due to negative feedback from the long standing hypercortisolaemia [17]. In our case, however, the resected adenohypophysis showed markedly widespread ACTH immunopositivity consistent with hyperplasia. In adults, corticotroph hyperplasia can very rarely be secondary to ectopic corticotropin-releasing hormone secretion [18]. In our case, however, the fact that the hyperplasia was focal, and that the patient went into remission following only partial resection of the pituitary makes secondary corticotroph hyperplasia very unlikely; ongoing close observation is, however, necessary.

Although cyclical hypercortisolism can be demonstrated in approximately 15 % of adult patients with Cushing’s disease, and perhaps up to 40 % of adults with Cushing’s syndrome [2, 19–21], it is much rarer in children, Only 2 cases were observed in a large case series of 59 paediatric patients with Cushing’s syndrome [22], and only one case in a large series that included 17 paediatric patients [2]. There are only ten reports of cyclical cortisol hypersecretion in the paediatric population in the literature [3]. These are predominantly due to nodular adrenocortical disease [22–24], although pituitary adenoma [19], ectopic corticotropin secretion [22], and unknown aetiology [25] have all been described. This therefore represents a very rare case of cyclical Cushing’s disease due to corticotroph hyperplasia in a paediatric patient. The demonstration of several late night salivary cortisol peaks and two normal troughs makes this a robust diagnosis of cyclicity.

Cushing’s disease is associated with a high rate of relapse, particularly in children [12], but early surgical remission rates following transsphenoidal surgery are comparable to those in the adult population [26]. The finding of severe postoperative hypocortisolism in the post-operative period indicates a good longer-term prognosis [11] (positive predictive value of 96 % for long-term remission in a paediatric population if postoperative morning cortisol < 28 nmol/L in one series). However, due to the cyclical nature of the original disease, close long-term follow-up, with repeated biochemical testing as well as clinical evaluation, will be essential for our patient. Most recently, the desmopressin test has been proposed as a useful predictor of recurrence of disease, either alone, or in combination with the dexamethasone test [27]. It is not, however, a well-established follow-up test for Cushing’s disease in the paediatric population, and we therefore do not have data on this.

This case illustrates the capricious nature of Cushing’s disease and demonstrates the necessity of both keeping a differential diagnosis in mind, and the utility of using different methods of assessing cortisol secretion in this diagnostically challenging condition.

## Consent

Written informed consent was obtained from the patient and his legal guardian for publication of this case report and any accompanying images. A copy of the written consent is available for review by the Editor of this journal.
